# Advantages and limitations of UV cross‐linking analysis of protein–RNA interactomes in microbes

**DOI:** 10.1111/mmi.15073

**Published:** 2023-05-10

**Authors:** Sofia Esteban‐Serna, Hugh McCaughan, Sander Granneman

**Affiliations:** ^1^ Centre for Engineering Biology, School of Biological Sciences University of Edinburgh Edinburgh UK

**Keywords:** microorganisms, protein‐RNA interactions, proteomics, RNA‐binding proteins, UV cross‐linking

## Abstract

RNA‐binding proteins (RBPs) govern the lifespan of nearly all transcripts and play key roles in adaptive responses in microbes. A robust approach to examine protein–RNA interactions involves irradiating cells with UV light to form covalent adducts between RBPs and their cognate RNAs. Combined with RNA or protein purification, these procedures can provide global RBP censuses or transcriptomic maps for all target sequences of a single protein in living cells. The recent development of novel methods has quickly populated the RBP landscape in microorganisms. Here, we provide an overview of prominent UV cross‐linking techniques which have been applied to investigate RNA interactomes in microbes. By assessing their advantages and caveats, this technical evaluation intends to guide the selection of appropriate methods and experimental design as well as to encourage the use of complementary UV‐dependent techniques to inspect RNA‐binding activity.

## Graphical abstract

UV light can be used to irreversibly join RNA‐binding proteins to the transcripts with which they interact. This process is called cross‐linking. By extracting the RNA of previously cross‐linked cells, it is possible to co‐purify all RNA‐binding proteins in a microbe. Mass spectrometry can then identify these proteins. Complementarily, a protein can be isolated with the RNAs to which it was cross‐linked. Sequencing of co‐partitioned transcripts informs about the RNA‐binding sites of a single protein.

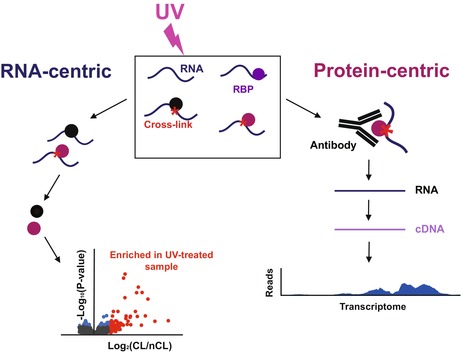

**Abbreviations**
2C: complex capture
AMT‐NHS: 4′‐aminomethyltrioxsalen linked to an N‐hydroxysuccinimide ester group
AzG: 2′‐deoxy‐2′‐azidoguanosine
CARIC: click chemistry‐assisted RNA‐interactome capture
cDNA: complementary DNA
CLASH: cross‐linking, ligation and sequencing of hybrids
CLIP: cross‐linking and immunoprecipitation
CRAC: cross‐linking and analysis of cDNAs
CRUIS: CRISPR‐based RNA‐united interacting system
dCLIP: denaturing CLIP
eCLIP: enhanced CLIP
FLASH: fast ligation of RNA after some sort of affinity purification for high‐throughput sequencing
FSY: fluorosulfate‐l‐tyrosine
GECX‐RNA: genetically encoded chemical crosslinking of proteins with RNA
GradR: gradient sedimentation with RNase treatment and mass spectrometry
Grad‐seq: gradient profiling by sequencing
hiCLIP: RNA hybrid and individual‐nucleotide resolution CLIP
irCLIP: infrared‐CLIP
IRP: iron regulatory protein
OOPS: orthogonal organic phase separation
PAR‐CLIP: photoactivatable ribonucleoside‐enhanced CLIP
PTex: phenol‐toluol extraction
RBP: RNA‐binding protein
RBPome: RNA‐binding proteome
R‐DeeP: RNA‐dependent proteins
RIC: RNA‐interactome capture
RICK: RNA interactome using click chemistry
RIL‐seq: RNA interaction by ligation and sequencing
RNP: ribonucleoprotein
RRM: RNA recognition motifs
RT: reverse transcriptase
SDA: succinimidyl 4,4′‐azipentanoate
SEC‐seq: size exclusion chromatography followed by RNA sequencing and mass spectrometry
SFY: o‐sulfonyl fluoride‐O‐methyltyrosine
ssRNA: single‐stranded RNA
STAMP: surveying targets by APOBEC‐mediated profiling
T4 PNK: T4 polynucleotide kinase
TRAPP: total RNA‐associated protein purification
TRIBE: targets of RBPs identified by editing
uvCLAP: ultraviolet crosslinking and affinity purification
XRNAX: protein‐cross‐linked RNA extraction

## 1 INTRODUCTION

From their synthesis to their degradation, RNAs are escorted by proteins that dictate their fate. In addition to transcription, interactions between RNA and proteins underlie post‐transcriptional processes, including RNA maturation, splicing, nuclear export, localisation, stability, translatability and degradation. RBPs fine‐tune gene expression profiles which are essential to maintain cellular homeostasis. Accordingly, disturbance of physiological RNA‐protein interactions decreases microbial capacity to rapidly reprogram the transcriptome and adapt to environmental changes (Chu et al., 2022; van Nues et al., 2017).

Methodological developments during the last decade have vastly increased the technical repertoire to explore RNA‐protein interactions in vivo. Most methods to detect RNA‐protein interactions are based on UV cross‐linking, which entails irradiating cells with short wavelength (254 or 365 nm) UV light to induce the formation of covalent bonds between RBPs and directly bound transcripts (“zero distance”; reviewed in Urdaneta & Beckmann, 2020). This property makes it possible to isolate RBP‐bound RNAs under very stringent and denaturing conditions, greatly reducing noise. Although UV cross‐linking is notoriously inefficient and biased towards coupling pyrimidines to a select number of amino acids (reviewed in Urdaneta & Beckmann, 2020), it has become a hugely popular tool to study protein‐nucleic acid interactions in living systems.

**FIGURE 1:**
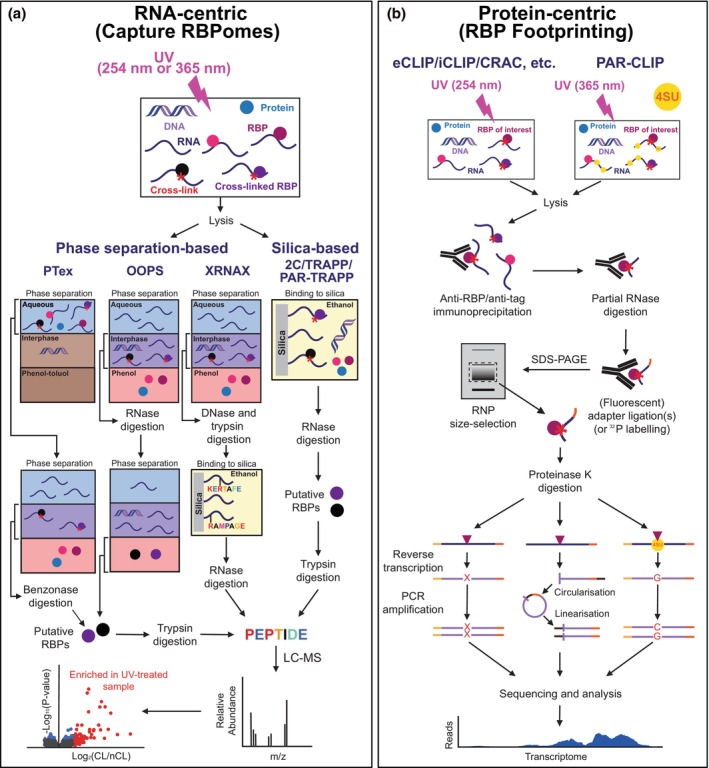
Diagrammatic overview of the RNA and protein‐centric approaches to study RNA‐protein interactions. (a) RNA‐centric methods (e.g., PTex, OOPS, XRNAX, 2C, TRAPP and PAR‐TRAPP) identify proteins binding to RNA (RBPs) in each system. PAR‐TRAPP uses uracil/uridine analogues (e.g., 4‐thiouracil; 4SU) to cross‐link proteins to RNA by UV irradiating cells at 365 nm. PTex and OOPS involve two rounds of organic extraction (i.e., phase separation) to purify cross‐linked RNPs. XRNAX uses a single round of organic extraction, followed by DNase treatment and trypsin digestion. Cross‐linked peptides can then be further purified using silica resin. (b) Protein‐centric techniques (e.g., CLIP, CRAC, iCLIP and PAR‐CLIP) output RNA‐binding footprints for RBPs. These protein‐centric approaches use stringent affinity purification methods to isolate the protein of interest and to enrich for the cross‐linked RNAs. PAR‐CLIP also employs uracil/uridine analogues (e.g., 4‐thiouracil) for protein‐RNA cross‐linking. Together these procedures allow the detection and functional characterisation of RNA‐binders in many organisms.

UV irradiation approaches used to study protein‐RNA interactions can be classified as RNA‐centric, which isolate RNA species to identify cross‐linked RBPs, or protein‐centric, which captures a specific RBP to study its bound RNA targets (Figure 1) (Ramanathan et al., 2019). Recently, a variety of UV‐based high‐throughput RNA‐centric strategies have characterised the RNA‐binding proteome (RBPome) in eukaryotic and prokaryotic microorganisms, which unearthed many novel RBPs (Asencio et al., 2018; Chu et al., 2022; Queiroz et al., 2019; Shchepachev et al., 2019; Urdaneta et al., 2019). Surprisingly, many of these newly identified proteins lack hitherto known RNA recognition motifs (RRM) or functional links to RNA metabolism. For instance, in multiple studies, metabolic enzymes constituted a prominent fraction of these putative RBPs (Hentze et al., 2018). However, each of these high‐throughput RNA‐centric approaches has its own technical caveats and noise levels. Therefore, protein‐centric procedures are critical to functionally validate recently discovered RBPs. By combining RNA and protein‐centric approaches one can shed light on how RNA binding affects (i) the life cycle of target transcripts and (ii) the primary function of the associated protein. For example, individual protein‐centric analyses have verified that, indeed, some metabolic enzymes moonlight as post‐transcriptional regulators (Huppertz et al., 2022). In fact, the catalytic activity of some of these non‐professional RNA binders, such as enolase, can be regulated by client transcripts which, in turn, can act as competitors for the enzyme's natural substrate (Huppertz et al., 2022).

This perspective article aims to offer a selective review of these protein and RNA‐centric options, discuss their individual strengths and limitations, consider possible technical improvements and present a complementary workflow for the identification and functional characterisation of novel RBPs in microorganisms.

## 2 DEFINING MICROBIAL RBPomes: PHASE‐SEPARATION VERSUS SILICA‐BASED STRATEGIES

The development of several RBPome profiling methods was inspired by well‐established whole‐cell RNA extraction protocols: the RNA‐interactome capture (RIC) procedure employs oligo‐dT beads which hybridised to polyadenylated transcripts (Castello et al., 2012). Since this technique specifically detects proteins bound to eukaryotic messenger RNAs, it is unsuitable to examine RBPs recognising transcripts lacking (long) poly‐A tails (e.g., eukaryotic ribosomal, transfer and small nucleolar RNAs as well as prokaryotic transcripts in general). However, this issue can be overcome by transiently overexpressing a poly‐A polymerase (Stenum et al., 2023). Click chemistry‐based methods (click chemistry‐assisted RNA‐interactome capture, CARIC; RNA interactome using click chemistry, RICK) have sought to circumvent this drawback by treating cells with synthetic uridine analogues (Bao et al., 2018; Huang et al., 2018). Once incorporated in newly transcribed RNAs, these compounds can be chemically linked to biotin using click chemistry and immunoprecipitated by streptavidin‐conjugated beads (Bao et al., 2018; Huang et al., 2018). Although click chemistry regents do not appear to alter cell viability (Huang et al., 2018), it is important to consider that these nucleotide analogues can be cytotoxic and alter the transcriptome. To address this, an innocuous click nucleoside analogue, 2′‐deoxy‐2′‐azidoguanosine (AzG) (Meng et al., 2020), was shown to be compatible with metabolic labelling in many bacteria. Despite making it possible to adapt CARIC/RICK‐based approaches to prokaryotes, AzG can only be incorporated into transcripts by a guanosine kinase, rendering this technique inapplicable to bacteria not encoding such enzyme. More recently, psoralen‐probes have also been successfully used for RNA tagging and subsequent RNP enrichment (Zhang et al., 2021). Nonetheless, given that psoralen‐derived compounds do not easily permeate cells, labelling is necessarily performed in lysates where psoralen, which presents a larger distance range than UV (Han et al., 2022), can incidentally bind to proteins in the surroundings.

Because of the limitations associated with RIC and RNA‐labelling methods for RBPome capture, several groups have explored alternative approaches. Gradient profiling by sequencing (Grad‐seq) established the earliest technical foundation for ribonucleoprotein (RNP) identification in bacteria in vivo (Gerovac et al., 2021; Smirnov et al., 2016). This method, which does not rely on UV, combines density gradient centrifugation with high‐throughput RNA sequencing and mass spectrometry to assemble transcript and protein inventories for each fraction. Subsequent grouping of RNA populations with matching sedimentation patterns identifies likely target cohorts that can be further verified by pull‐down assays in a few transcripts of each population. Ongoing methodological advances have expanded on this concept to give rise to SEC‐seq (size exclusion chromatography followed by RNA sequencing and mass spectrometry) (Chihara et al., 2022), which pairs high‐resolution size exclusion chromatography with downstream transcriptomic clustering and bait‐based validation. Apart from presenting higher resolution and applicability to physiological contexts, SEC‐seq has paved the development of techniques such as R‐DeeP (RNA‐dependent proteins) or GradR (gradient sedimentation with RNase treatment and mass spectrometry) which have incorporated an RNase treatment step to facilitate gradient‐based partitioning of candidate RBPs (Caudron‐Herger et al., 2019; Gerovac et al., 2020). Overall, these unbiased approaches are producing hugely valuable data leading to, for example, the identification of a key bacterial RNA chaperone (Smirnov et al., 2016). But, since they do not allow recognition of RBPs in direct association with RNA, UV cross‐linking‐based techniques could be highly complementary (Table 1). In this respect, the first UV‐derived snapshots of bacterial RBPomes arrived with the advent of the orthogonal organic phase separation (OOPS) and phenol–toluol extraction (PTex) protocols, which phase‐extract molecules depending on their physicochemical properties (Figure 1) (Queiroz et al., 2019; Urdaneta et al., 2019). Concurrently, the protein‐cross‐linked RNA extraction (XRNAX) approach employed a similar basis in human cells (Trendel et al., 2019).

**TABLE 1:** Summary of the methods mentioned throughout the text.

UV	Name	Summary	Considerations
UV‐dependent	RNA‐centric (i.e., for RBPome capture)		
	OOPS (Queiroz et al., 2019)	Phase‐extract RNPs based on their physicochemical properties	Despite requiring minimal starting material, these techniques cannot be used to examine the RNA‐binding activity of molecules with similar physicochemical characteristics to those of RNPs
	PTex (Urdaneta et al., 2019)		
	XRNAX (Trendel et al., 2019)	Separates proteins from other cellular components using phenol extraction and further enriches for RNPs during a silica‐based titration step	Introducing a silica‐dependent purification step reduced noise in the resulting profile. However, this method has only been applied to mammalian cells so far
	2C (Asencio et al., 2018)	Isolate RNPs using silica‐conjugated platforms and ethanol washes	Simple and quick. While they yield a higher number of enriched RBPs, these techniques generally require a larger number of input cells. In some instances, the number of false positives can be higher
	TRAPP (Shchepachev et al., 2019)		
	PAR‐TRAPP (Shchepachev et al., 2019)		
	Protein‐centric (i.e., for RBP footprinting)		
	CLIP (Ule et al., 2003)	Immunoprecipitates the RBP of interest under semi‐denaturing conditions, trims unshielded ribonucleotide sequences, ligates adapter sequences and digests the RBP from the purified RNPs. Upon reverse transcription, sequencing of the resulting cDNA library enables transcriptome‐wide identification of the sites to which the tested RBP binds	Original protocol. Relies on the base substitutions introduced by the reverse transcriptase to map cross‐linking sites
	iCLIP (Huppertz et al., 2014)	CLIP‐based protocol. Incorporates the sequencing adapter to the 5′ end of the reverse transcription primer, circularises the cDNA and re‐linearises the molecule to leave the adapter in the 3′ end of the cDNA. Importantly, this allowed library construction from prematurely terminated cDNAs	Maps cross‐linking sites at single‐nucleotide resolution based on cDNA truncations
	eCLIP (Van Nostrand et al., 2017)	CLIP‐based protocol. Introduces a DNA adapter ligation step after reverse transcription. It was proposed that this enhances adapter ligation by around 1000‐fold	Forgoes RNPs visualisation which generally aids experimental optimisation, detection, and removal of contaminants
	irCLIP (Zarnegar et al., 2016)	CLIP‐based protocol. Ligates DNA adapters conjugated to an IR800CW fluorescent dye	Avoids radioactive RNA labelling but still resolves RNPs by PAGE. It is unclear whether fluorescence‐based approaches are more sensitive than RNP detection using radioactivity
	easyCLIP (Porter et al., 2021)	CLIP‐based protocol. Employs fluorescent adapters to quantify cross‐linked RNAs	Discriminates between RBPs and proteins lacking RNA‐recognition capacity and allows visualisation of ligation efficiency
	PAR‐CLIP (Hafner et al., 2010)	CLIP‐based protocol. Supplements cells with photoactivable 4‐thiouracil or 6‐thioguanine to enhance the cross‐linking efficiency and monitors T‐to‐C or G‐to‐A substitutions as an indicator of RNA cross‐linking	Prolonged exposure to the nucleobase analogues can be cytotoxic. Additionally, metabolic RNA labelling methods have been difficult to implement in prokaryotic organisms. It is unsuitable for tissues
	CRAC (Granneman et al., 2009)	CLIP‐based protocols. Apply (tandem) affinity‐based chromatography to separate RBPs based on specific epitope tags. This partition approach is compatible with denaturing purification and the use of strong detergents	Tagging can affect the stability, expression, and RNA‐binding activity of the RBP under study. Nevertheless, the additional purification steps can significantly decrease background noise
	uvCLAP (Maticzka et al., 2018)		
	dCLIP (Rosenberg et al., 2017)		
	FLASH (Ilik et al., 2019)		
	CLASH (Kudla et al., 2011)	CLIP‐based protocols. Use RBPs harbouring RNA–RNA interactions as baits to map and functionally characterise non‐coding transcripts	Are subject to identification and selection of bona fide RNA–RNA chaperones
	hiCLIP (Sugimoto et al., 2015)		
	RIL‐seq (Melamed et al., 2016)		
UV‐independent	RNA‐centric (i.e., for RBPome capture)		
	RIC (Castello et al., 2012)	Polyadenylated transcripts are purified using oligo‐dT beads	Inapplicable to prokaryotic transcripts unless oligo‐A tails are added artificially
	CARIC (Huang et al., 2018)	Treats cells with nucleoside analogues that can be chemically linked to biotin. Subsequently, RNP immunoprecipitation is performed with streptavidin beads	Restricted to newly synthesised transcripts. Moreover, supplementing nucleoside analogues can diminish cell viability
	RICK (Bao et al., 2018)		
	AzG RNA Metabolic Labelling (Meng et al., 2020)		Restricted to nascent transcripts and dependent on existence of a native guanosine kinase
	Grad‐seq (Smirnov et al., 2016)	Combines density gradient centrifugation with RNA sequencing and mass spectrometry to assemble transcript and protein catalogues for each fraction	Do not capture RBPs in direct association with RNA
	R‐DeeP (Caudron‐Herger et al., 2019)	Based on Grad‐seq. Add an RNase digestion step to facilitate gradient‐based partitioning of candidate RBPs	
	GradR (Gerovac et al., 2020)		
	SEC‐seq (Chihara et al., 2022)	Pairs high‐resolution size exclusion chromatography with downstream transcriptomic clustering and bait‐based validation	
	GECX‐RNA (Sun et al., 2023)	Replaces some residues in the RBP of interest with synthetic amino acids that unbiasedly interact with ribonucleotides in their close surroundings	Amino acid substitutions can affect the structure, function, and RNA‐recognition capacity of the protein
	CRUIS (Zhang et al., 2020)	Targets a specific transcript using CRISPR and simultaneously tags interacting RBPs with a proximity‐labelling enzyme	Chemically modifying an RBP can disrupt its physiological activity, stability, and RNA‐binding affinity
	Protein‐centric (i.e., for RBP footprinting)		
	TRIBE (Brannan et al., 2021)	Couples RBPs to RNA‐editing enzymes that insert nucleotide modifications used as a proxy for RNA binding events	Can impair the expression and function of the RBP under study and induce cytotoxicity by uncontrolled transcriptomic hyperedition
	STAMP (McMahon et al., 2016)		

*Note*: Techniques have been classified according to their UV dependence and experimental principle. A brief review of key features of each procedure is offered in the last column.

OOPS applies identical principles to those of standard acid guanidinium thiocyanate‐phenol‐chloroform RNA extraction procedure. Briefly, upon detergent and phenol addition to the aqueous phase, denatured proteins and lipids partition to the hydrophobic phenol phase and free RNA molecules migrate to the upper water‐rich layer. Due to their intermediate solubility in these conditions, DNA and RNA‐protein adducts are predominantly found in the interphase. After trimming the RNA of the RNP complexes in the interphase with RNases, the RBPs can subsequently be recovered in downstream phenol extraction rounds from the organic phase. In turn, performing an initial pheno‐toluol phase separation at neutral pH, PTex exploits toluol's higher insolubility and lower density to separate DNA and lipids, which shift to a phenol‐based organic interphase, from soluble RNA, proteins and covalently linked RNPs in the aqueous phase. Following chaotropic treatment of the aqueous phase, phenol addition confines unfolded proteins and unbound RNA to the organic and aqueous phases, respectively, and, ultimately, allows direct RBPs precipitation from the interphase fraction.

### 2.1 Organic phase extraction versus silica‐based purification

Compared to previous strategies, methods built upon consecutive phase extractions are independent of the sequential features of cognate RNAs and suitable to study the RBPome in prokaryotes such as *Escherichia coli*, *Salmonella enterica* and *Staphylococcus aureus* (Chu et al., 2022; Queiroz et al., 2019; Urdaneta et al., 2019). Moreover, in contrast to titration‐based techniques, phase partition methods require the lowest number of input cells for putative RBP identification. However, phenol separation can be technically challenging to perform without residual spillover between fractions, which may result in higher background contrasted with some other protocols if not performed carefully. Furthermore, it has been reported that some stable RNPs can be recovered from interphase fractions even without UV irradiation (Trendel et al., 2019). Organic phase extraction protocols are also inherently incapable of detecting RNA‐binding activity in molecules with similar characteristics to those of RNPs; namely, glycoproteins (Smith et al., 2020). Nevertheless, a significant advantage of employing organic phase extraction approaches is that they also enrich for cross‐linked RNAs, making it possible to globally identify protein‐binding sites within RNAs (Queiroz et al., 2019; Urdaneta et al., 2019).

To complement the phase partition protocols, several studies have employed silica‐based RNA‐centric approaches. Positively charged silica matrices are routinely used to isolate nucleic acids by interacting with the phosphate groups in their backbone. Under stringent conditions, proteins are weakly bound to silica surfaces and, consequently, they are easily removed by washes. Importantly, recent work discovered that silica's charge‐based interaction with polynucleotides was strong enough to retain RNA‐protein adducts in human and yeast lysates (Asencio et al., 2018; Shchepachev et al., 2019). As ethanol favours RNA adsorption over DNA binding (Avison, 2006), the complex capture (2C) and total RNA‐associated protein purification (TRAPP and PAR‐TRAPP) procedures have coupled this isolation protocol to liquid chromatography with tandem mass spectrometry (LC–MS/MS)‐based proteomics (Figure 1) (Asencio et al., 2018; Shchepachev et al., 2019). Subsequently, these techniques have offered simple and quick alternatives for RBPome profiling in human cell lines, *Saccharomyces cerevisiae*, *E. coli* and *S. aureus* (Asencio et al., 2018; Chu et al., 2022; Shchepachev et al., 2019). It would be interesting to combine Grad‐seq and SEC‐seq with RBPome indexing methods as we envision that the fractionation of the cell lysates would further increase the sensitivity of RBPome approaches. As an illustration, UV treating the cells before performing density gradient ultracentrifugation or size exclusion chromatography, followed by RBPome analysis of individual fractions would make it possible to identify which proteins in RNPs detected in Grad‐seq/SEC‐seq fractions likely bind directly to RNA.

### 2.2 Dealing with noise

In contrast to organic phase extraction approaches, silica‐based strategies typically yielded higher numbers of significantly enriched RNA binders. In fact, whereas prior reports had estimated RBPs to conform around a tenth of eukaryotic and bacterial proteomes (Castello et al., 2012; Queiroz et al., 2019), silica‐based findings consistently record portions between 20% and 30% (Chu et al., 2022; Shchepachev et al., 2019). It is plausible that spurious proteins co‐purify with RNPs if, like histones or nucleotide‐binding enzymes, they bind to DNA or short nucleotides (Stützer et al., 2020). However, many of these proteins were also abundantly detected in datasets stemming from organic phase separation experiments, which cannot detect RNA‐binding in transcripts shorter than 30 nucleotides in length (Urdaneta et al., 2019). Regardless, we strongly recommend testing a range of UV irradiation treatments and selecting the lowest possible dose when studying protein‐RNA interactions (Shchepachev et al., 2019): high levels of UV not only cause substantial protein and RNA degradation (Chu et al., 2022; McKellar et al., 2020) but also can cross‐link proteins to DNA (Stützer et al., 2020). Thus, treating lysates with DNase is also advisable. Notably, although 2C and TRAPP indeed generate larger pools of statistically enriched proteins, they generally require a larger number of input cells to do so. This constitutes a technical cost for the poorer binding capacity of silica‐based capture compared to self‐contained phase separation systems.

Despite the obvious methodological differences as well as the advantages and shortcomings which they prompt, silica‐dependent and organic phase separation‐based techniques produced remarkably similar results for *S. aureus* (Chu et al., 2022). Together with individual validation of the RNA‐interaction status of some new RBPs, this first direct comparison underscored that both approaches are fit for holistic interrogation of microbial RBPomes. In fact, successively applying the purification principles of both strategies could improve the current individual performance of both methods. For instance, in the XRNAX protocol, mammalian RNPs were coarsely partitioned from other cellular components using phenol extraction. After trypsin‐digesting the recovered proteins, the cross‐linked peptides were subsequently enriched using a silica‐conjugated platform, which contributed to reduce noise (Figure 1) (Trendel et al., 2019).

## 3 PROTEIN‐CENTRIC APPROACHES: CROSS‐LINKING AND IMMUNOPRECIPITATION

Even though detection of RBPs in datasets derived from several RNA‐centric RBPome studies is frequently a reliable indicative of RNA recognition, candidate RBPs should not be considered bona fide RNA binders until they have been shown to bind RNA in their native systems. Ideally, this should be done using a variety of orthogonal in vitro and in vivo methods. The discovery of non‐canonical RNA‐binders among some metabolic enzymes or DNA‐binding proteins emphasises the importance of verifying direct ribonucleotide binding. This validation is essential to exclude false positives that may have been introduced by experimental artefacts. Furthermore, protein‐centric studies can provide pivotal evidence for determining the physiological role of such RNA‐binding events.

The most widely used techniques for globally identifying the RNAs bound to RBPs are cross‐linking and immunoprecipitation (CLIP) and related protocols, such as CRAC, eCLIP, iCLIP and PAR‐CLIP (Granneman et al., 2009; Hafner et al., 2010; Huppertz et al., 2014; Ule et al., 2003; Van Nostrand et al., 2016). Like RBPome capture, these approaches rely on UV cross‐linking of RBPs to their target transcripts. To enrich for the cross‐linked protein (and, therefore, the RNAs to which it is likely to bind directly), immunoprecipitations are generally performed under (semi‐)denaturing conditions (Granneman et al., 2009; Hafner et al., 2010; Ule et al., 2003; Van Nostrand et al., 2016). Following RNase trimming of unshielded ribonucleotide sequences, adapter ligation and proteinase K digestion of the preserved RNPs, high‐throughput sequencing allows transcriptome‐wide mapping of the sites to which the tested RBP was binding (Figure 1). Despite its technical power and widespread use, CLIP presents some limitations. A family of CLIP methods has emerged to provide solutions for some of these challenges in specific biological contexts (Table 1) (Lee & Ule, 2018).

### 3.1 Optimising CLIP for individual RBPs and including appropriate controls

A challenge of CLIP procedures is, again, their dependence on UV exposure to generate RNA‐protein adducts. As outlined above, in vivo UV radiation is particularly inefficient: it usually takes minutes to complete and only about 1%–5% of RNPs of interest are cross‐linked (Darnell, 2010). Hence, some CLIP variants often require a relatively large number of cells. Systematically poor cross‐linking performance could be attributed to UV preferences for generating duplexes between pyrimidines and aromatic residues, its bias towards covalently linking stacking interactions, its inability to create adducts with some amino acids or its intrinsically limited penetration in certain cell types or growth media (Chu et al., 2022; Knörlein et al., 2022; Urdaneta et al., 2019). Recently, we developed a much‐improved UV cross‐linker (Vari‐X‐linker; UVO3) (McKellar et al., 2020; van Nues et al., 2017) that greatly increases the cross‐linking efficiency in actively growing cells during markedly shorter time spans (seconds) (McKellar et al., 2020; van Nues et al., 2017). Still, the fastest and most efficient way to cross‐link proteins to RNA is using UV lasers (Sharma et al., 2021), although setting up such a system can be prohibitively expensive. Another major advantage of these rapid cross‐linking devices is that they enable the monitoring of very dynamic changes in protein–RNA interactions, such as those occurring during stress responses, at high temporal resolution (Bresson et al., 2020; McKellar et al., 2020; Sharma et al., 2021; van Nues et al., 2017).

**FIGURE 2:**
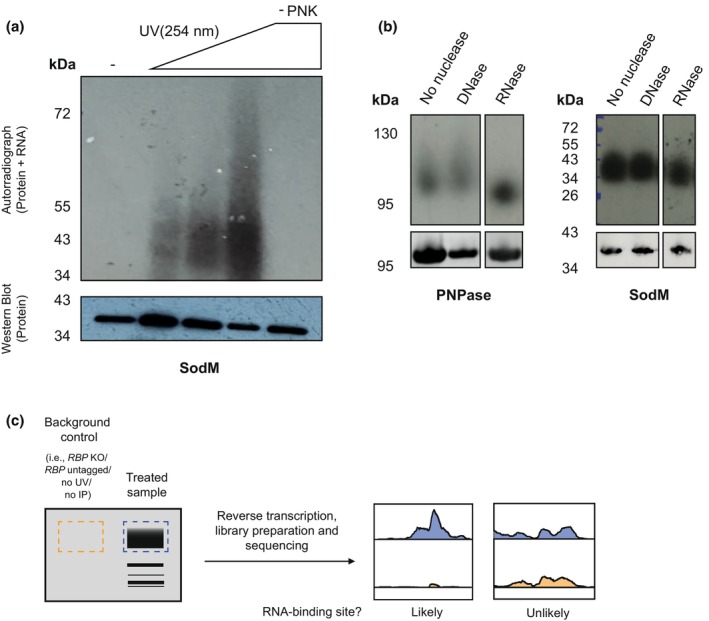
Good practices for CLIP and related methods. (a) Testing a range of UV exposure times is recommended to find the minimal dose at which enough molecules of the RBP of interest are cross‐linked to their RNA targets. No visible signal should be observed when the T4 PNK treatment is omitted as radiolabelled phosphate groups would not be added to the transcripts in the RNPs. (b) The migration pattern of an RNP (e.g., PNPase) should display a smear reflecting the different RNAs to which the RBP is bound. Accordingly, the signal should be unaffected by DNase treatment. However, digestion of cross‐linked RNPs with RNases should trim bound transcripts to a length corresponding to the part of the sequence that is shielded by the RBP during digestion. Consequently, the signal of a professional RBP resolves upon RNase treatment. Still, it is possible that some unconventional RBPs (e.g., SodM) could be bound to small transcripts and, thus, remain unaffected by treatments with nucleases. (c) Sequencing cDNA libraries derived from control samples allows quantification of background signal and the subsequent identification of real RNA binding sites. Control samples could comprise a strain in which the gene of interest is knocked‐out, an untreated sample, a CLIP experiment without antibodies or unrelated ones or, in the case of CRAC, a strain where the protein of interest is untagged.

To quantify RNA‐protein cross‐linking efficiencies, most protocols attach radioactive phosphates or fluorescently labelled oligonucleotides to 5′ ends of the cross‐linked transcripts. The RNP complexes are subsequently resolved by denaturing PAGE (Figure 2) and the RNA can then be visualised by autoradiography or fluorescent imaging. Recently, we performed such radiolabelling analyses on a variety of RBPs identified in our *S. aureus* RBPome analyses (Chu et al., 2022). While the vast majority of the proteins tested detectably cross‐linked to RNA, we learned that only for those proteins for which we observed very strong radioactive signals (1 to 3‐h exposure of phosphorimager screen or autoradiography film) were we able to obtain high‐complexity complementary DNA (cDNA) libraries (Chu et al., 2022). It is also important to point out that the radiolabelling assay can generate false positive signals: radioactive labelling of cross‐linked RNA involves an incubation step with T4 polynucleotide kinase (T4 PNK). Hence, it is possible that during this reaction, the RBP of interest becomes radiolabelled by autophosphorylation or co‐purified host kinases (Tawk et al., 2017). Consequently, we would always advise including control reactions with non‐irradiated cells or leaving out PNK altogether (Figure 2a).

As well as informing the choice of UV exposure, preliminary signal evaluation provides a strategy by which to filter out proteins with no or little RNA‐binding activity. As outlined above, UV irradiation can also cross‐link proteins to DNA (Stützer et al., 2020). Accordingly, we strongly recommend performing control experiments where the cross‐linked RNPs are incubated with increasing concentration of RNase I/A or DNase I. If the purified RNP indeed contains cross‐linked RNA, higher concentrations of RNase should reduce the smearing of the bands corresponding to RNPs (Figure 2b; PNPase). Alternatively, the intensity of those containing DNA‐protein duplexes would only be resolved by DNase digestion. We also found that not all cross‐linked proteins, such as the *S. aureus* superoxide dismutase SodM, respond to either DNase or RNase treatment (Figure 2b). This suggests that these proteins generally bind short nucleic acid fragments, which will make the library preparation steps more challenging.

Being a zero‐distance cross‐linking method (Urdaneta & Beckmann, 2020), UV irradiation minimises the risks of generating unspecific contacts. Yet, especially when studying non‐professional RBPs, it is possible to get systemic and reproducible RNA signals that are not proceeding from genuine binding. In instances where test CRAC experiments are inconclusive (Figure 2a; SodM), we would recommend contextualising putative ribonucleotide recognition within the main function of the protein. As mentioned above, the enzymatic activity of some non‐professional RBPs conditions their capacity to bind their RNA ligands because transcripts could be competing with their primary substrate. More precisely, in the case of SodM, it would be advisable to examine the variation of the signal in conditions where the activity of the enzyme is maximised, such as in the presence of iron or manganese, or when its catalytic action is metabolically (e.g., during iron starvation) or genetically prevented (i.e., in a mutant strain with abolished iron or manganese binding sites). In fact, a similar experimental rationale was followed to assess the RNA‐binding capacity of the iron regulatory protein (IRP) in human cells (Khan et al., 2017; Pantopoulos et al., 1995).

### 3.2 Recent improvements to the CLIP protocol

Validating RNA recognition by a conserved protein across species constitutes powerful evidence to ascertain biologically meaningful RNA‐binding activity. In microbes, this strategy was pioneered by a comparative analysis of the client transcripts of ProQ in *E. coli* and Salmonella grown under identical conditions (Holmqvist et al., 2016, 2018). The first of these studies also introduced a binding site detection algorithm specifically suited for identifying RNA‐binding sites for prominent bacterial RBPs (Holmqvist et al., 2016). Until then, available software merged all sites to fit a background model above which significance is eventually called. Nevertheless, this approach imposed inadequate constrains when studying ubiquitous RNA binders in prokaryotic organisms with smaller genomes. Hence, to compensate for (i) the lack of a standardised control sample selection for CLIP‐based protocols and (ii) the fact that the abundance of a given RNA ligand will unavoidably affect its read coverage in the resulting cDNA library irrespectively of the affinity of the RBP for that target, this tool performed statistical tests on binding regions considering their local RNA counts (Holmqvist et al., 2016).

Besides computational advancements, the last decade has witnessed the dawn of faster, simpler and more efficient versions of the CLIP protocol. Since these have already been thoroughly reviewed in two recent publications (Hafner et al., 2021; Lee & Ule, 2018), we will not focus on them exhaustively and solely outline some of these developments. The most salient of these methods is the enhanced CLIP (eCLIP) procedure, which, instead of circularising and linearising cDNAs for multiplexing, introduced a 3′ DNA adapter ligation step succeeding reverse transcription (Figure 1) (Van Nostrand et al., 2016). Remarkably, this technical modification increased adapter ligation efficiency by approximately 1000‐fold and, therefore, greatly reduced the cellular input material required for the successful mapping of RNA‐binding sites (Van Nostrand et al., 2016). Nonetheless, eCLIP omitted RNP size evaluation, which, in turn, can guide methodological optimisation (Figure 2), spot contamination and assess the quality of the experiment.

Because radioisotope‐reliant visualisation is cumbersome to execute and requires specialised radiation rooms, more recent CLIP protocols are employing fluorescent labelling of cross‐linked RNAs as an alternative for visualising cross‐linked RBPs. For example, infrared‐CLIP (irCLIP) employs DNA adapters conjugated to an IR800CW dye (Zarnegar et al., 2016). Expanding on this principle, easyCLIP, has been able to estimate absolute RNA quantities from fluorescent visualisation and define a cross‐link rate threshold that can discern between candidate proteins with actual RNA‐binding activity and proteins lacking RNA‐recognition capacity (Porter et al., 2021). Crucially, this technique will facilitate preliminary examination of the many putative RBPs that have been detected by RNA‐centric techniques as it represents a streamlined informative test that could be run before undertaking a full CLIP‐type experiment. Another major improvement introduced by easyCLIP is the possibility of directly visualising 5′ and 3′ adapter ligation efficiencies using different infrared dyes (Porter et al., 2021). Importantly, this feature allows users to determine how efficient the ligation reaction for individual linkers was, which was previously not possible.

### 3.3 Mapping the UV cross‐linking sites in RNA

To be able to sequence the cross‐linked RNAs, they need to be converted into cDNAs. For this purpose, the cross‐linked protein is digested with proteinase K. However, this treatment does not remove amino acids cross‐linked to the RNA, which can significantly impact the processivity of the reverse transcriptase (RT) during cDNA synthesis. The cross‐linked amino acid residues generally cause the RT enzyme to drop off (Urlaub et al., 2002), yielding truncated cDNAs (Figure 1, iCLIP (Huppertz et al., 2014)). Nevertheless, the reverse transcriptase can also introduce mutations in cDNAs at the site of cross‐linking (Figure 1, CRAC (Granneman et al., 2009)) and continue to reverse transcribe the whole RNA fragment. Readthrough occurrences can be favoured by replacing magnesium with manganese in a reaction catalysed by a highly processive RT, namely Superscript IV (Van Nostrand et al., 2017). UV cross‐linking sites on the RNA can subsequently be determined at nucleotide resolution by mapping the nucleotide positions where the RT fell off, or by mining the data for mutations within reads. Alternatively, photoactivatable ribonucleoside‐enhanced CLIP (PAR‐CLIP) uses photoactivable 4‐thiouracil or 6‐thioguanine to enhance the cross‐linking efficiency and use T‐to‐C or G‐to‐A substitutions as a proxy for RNA cross‐linking (Figure 1) (Hafner et al., 2010). Conveniently, the PAR‐CLIP protocol has been modified for some microorganisms, such as the yeasts *S. cerevisiae* and *Schizosaccharomyces pombe* (Schaughency et al., 2014; Wittmann et al., 2017), where the technique is now routinely applied. However, adapting such metabolic labelling methods to prokaryotic model systems has proved to be significantly more challenging (Meng et al., 2020), which may explain why (at the time of writing) only a single manuscript describes the application of PAR‐CLIP in bacteria (Ojha & Jain, 2023). Additionally, prolonged exposure to the mentioned nucleobase analogues can cause cellular toxicity (Huppertz et al., 2014; Meng et al., 2020).

### 3.4 Dealing with experimental noise

The applicability of CLIP‐derived procedures is contingent to the extent of their background. A general assumption of these protocols is that random binding is hampered by the harsh washing steps succeeding immunoprecipitation. Moreover, electrophoretic resolution of the RNPs under study ensures an additional enrichment step with respect to adventitiously co‐partitioned RBPs. Preferred control samples have normally been acquired from cells where the gene encoding the RBP of interest is knocked out, untagged or which have not been UV treated (Figure 2b,c) (Granneman et al., 2009; König et al., 2010; Lee & Ule, 2018; Ule et al., 2003). In principle, these samples are not expected to produce any noticeable signal upon SDS‐PAGE visualisation. Consequently, libraries emerging from the sequences in the areas corresponding to the expected migration of the RNP under study should contain 100‐fold less unique cDNAs than those resulting from fully treated samples (König et al., 2010). Nonetheless, meta‐analysis of 30 published CLIP datasets identified widespread and replicable background reads across most of them (Friedersdorf & Keene, 2014). To quantify technical background, prior work sequenced libraries proceeding from RNA‐protein adducts that did not migrate with the duplexes of interest (Friedersdorf & Keene, 2014). Adjusting output signals to those originated by background characterisation was shown to dramatically improve the identification of RNA recognition sequences (Friedersdorf & Keene, 2014). Based on relevant control samples, background correction can indeed statistically disregard binding events resulting from non‐specific transcript cross‐linking to incidentally proximal RBPs of interest, co‐purified RNPs and RNA aptamers adopting an epitope‐mimicking conformation.

A number of CLIP‐related approaches have been specifically designed to further improve signal‐to‐noise ratios. These methods take full advantage of the fact that cross‐linked RNPs can withstand highly denaturing purification conditions. These techniques include cross‐linking and analysis of cDNAs (CRAC), ultraviolet crosslinking and affinity purification (uvCLAP), denaturing CLIP (dCLIP) and “Fast Ligation of RNA After Some sort of affinity purification for High‐throughput sequencing” (FLASH) (Granneman et al., 2009; Ilik et al., 2019; Maticzka et al., 2018; Rosenberg et al., 2017). All of these protocols employ (tandem) affinity‐based purification to recognise specific epitope tags that are compatible with denaturing purification conditions and/or strong detergents (e.g., HIS‐tag and streptavidin‐binding peptides sequences; Figure 1) (Granneman et al., 2009; Maticzka et al., 2018; Rosenberg et al., 2017). Admittedly, epitope tagging can alter the stability, expression and RNA‐binding capacity of the RBP of interest. Advantageously, however, specific tag recognition‐based partition spares the need for high‐quality antibodies, which are not readily available for less well‐studied model organisms. Accordingly, these approaches can considerably reduce background reads in resulting cDNA libraries. This property also makes them good alternatives to inspect scarcer RNPs, namely yeast pre‐ribosomal complexes or those encompassing non‐professional RBPs (Chu et al., 2022; Granneman et al., 2009).

## 4 ALTERNATIVES TO UV IRRADIATION

Although beyond the scope of this article, we feel that it is important to highlight recent approaches that have striven to surmount the issues associated with using UV to study protein‐RNA interactions (Table 1). On the one hand, techniques such as TRIBE (targets of RBPs identified by editing) or STAMP (surveying targets by APOBEC‐mediated profiling) fuse RBPs of interest to RNA‐editing enzymes that introduce nucleotide modifications as a proxy for RNA binding events (Brannan et al., 2021; McMahon et al., 2016). Complementarily, CRUIS (CRISPR‐based RNA‐united interacting system) targets individual RNA species using CRISPR and simultaneously tags interacting RBPs with a proximity‐labelling enzyme (Zhang et al., 2020). While these strategies minimise handling and input amounts, genetically or chemically tagging an RBP could jeopardise its physiological activity and expression or, when a modifying enzyme is attached to it, induce cytotoxicity by uncontrolled transcriptomic hyperedition.

On the other hand, proteins can also be chemically cross‐linked to RNA. Traditionally, formaldehyde is used for this purpose (Patton et al., 2020), but this treatment also links proteins to other proteins and DNA nucleotides located within 2 Å (Patton et al., 2020; Ramanathan et al., 2019). As a result, several groups have begun to develop more specific chemical cross‐linkers that appear to have higher cross‐linking efficiencies compared to UV (e.g., succinimidyl 4,4′‐azipentanoate (SDA) and AMT‐NHS, a psoralen‐derivative 4′‐aminomethyltrioxsalen (AMT) linked to an N‐hydroxysuccinimide ester group (NHS) (Han et al., 2022; Weidmann et al., 2021)). Yet, a limitation of these reagents is that they only react with lysine residues and, therefore, these chemicals do not fully circumvent the biases associated with UV irradiation. To overcome nucleotide preferences, the genetically encoded chemical crosslinking of proteins with RNA (GECX‐RNA) approach replaces some residues in the RBP under study with unnatural amino acids, namely fluorosulfate‐L‐tyrosine (FSY) and o‐sulfonyl fluoride‐O‐methyltyrosine (SFY), which uniformly react with ribonucleotides in their vicinity (Sun et al., 2023). However, introducing amino acid substitutions in an RBP can affect its structure, function and RNA‐recognition capacity. Regardless, these works represent a significant advancement in the field, and we hope they will encourage the development of a wider array of chemical cross‐linkers (Han et al., 2022; Sun et al., 2023; Weidmann et al., 2021).

Unlike UV, which tends to yield RNA‐protein duplexes enclosing single‐stranded RNA (ssRNA) species, chemical cross‐linkers reportedly display unbiased adduct formation patterns for single‐ and double‐stranded RNA targets (Han et al., 2022). We foresee that this feature will nurture future technical advances to investigate RNA–RNA interactions. To date, even despite its ssRNA bias, UV cross‐linking has enabled successful retrieval of RNA–RNA associations from immunoprecipitated RBPs harbouring such interactions. Soon after being identified as a suitable tool to analyse ncRNA‐mRNA interactions from yeast RNPs (Granneman et al., 2009), CRAC and other existing CLIP‐based protocols were amended to favour intermolecular ligation of complementary RNA species in RNA–RNA base‐pairing hubs (Kudla et al., 2011). Resulting methods, such as CLASH (cross‐linking, ligation and sequencing of hybrids), hiCLIP (RNA hybrid and individual‐nucleotide resolution CLIP), and RIL‐seq (RNA interaction by ligation and sequencing) (Kudla et al., 2011; Melamed et al., 2016; Sugimoto et al., 2015), have used RBPs chaperoning RNA–RNA interactions as bait to map and functionally characterise non‐coding transcripts (Table 1). So far, these procedures have been used in systems including mammalian cells, *Clostridioides difficile*, *E. coli*, Salmonella, *S. aureus* and *Vibrio cholerae* (Fuchs et al., 2022; Helwak et al., 2013; Huber et al., 2022; Matera et al., 2022; McKellar et al., 2022; Mediati et al., 2022; Melamed et al., 2016, 2020; Pearl Mizrahi et al., 2021; Sugimoto et al., 2015; Waters et al., 2017), which unravelled large ncRNA–RNA interactomes in these organisms. Eventually, customising present protocols to replace UV irradiation by chemical cross‐linking could verify and expand prevailing knowledge on the microbial and metazoan RNA–RNA interactome.

## 5 CONCLUSION

UV cross‐linking lies at the core of several techniques to study RNA interactions. Subsequent formation of covalent bonds ensures maintenance of the complex and facilitates isolation of RNPs. Purifying the transcriptome to capture protein‐RNA interactions has spurred the characterisation of RBPomes in an increasing number of microorganisms. Inevitably, the chance of recovering enriched adducts between proteins and hypothetical transcript targets are higher when whole‐cell RNA species are used as partitioning baits. Since transcriptome‐wide indexing of RBPs is more likely to yield false positives, it is crucial to validate putative RNA‐binding activity using protein‐centric approaches.

The advantage of producing validatory RNA maps is two‐fold. Firstly, interpreting RBP binding profiles can help to unravel their role and its underlying mechanism. Secondly, considering that harsher purification is generally possible in UV‐requiring methods, these tend to produce less background than their non‐UV‐dependent counterparts. However, cross‐linking efficiency and biases may result in false positive signals arising from the phosphorylation of DNA or the RBP itself. Furthermore, it is also plausible that the abundance, localisation or function of a given protein foster consistent but inconsequential RNA cross‐linking. As discussed, these situations should become apparent in appropriate downstream functional explorations. Nevertheless, including the mentioned control experiments could economise the efforts invested in characterising proteins which were circumstantially forming duplexes with RNA in previous datasets.

Methodological expansion of these protocols could provide insight into the biology of other components of the RNA interactome. As an illustration, we have referred to CLASH and related protocols, which have specified several RNA–RNA interactions as well as their regulatory functions. Despite not having been reviewed here, an evolving array of techniques has also been using cross‐linking and immunoprecipitation to study how the distribution of RNA modifications shape RBP occupancy (Hussain et al., 2013; Schwartz et al., 2013). Ultimately, this interplay of experimental tools will empower a confident understanding of protein‐RNA interactions and their implications in wider biological systems.

## AUTHOR CONTRIBUTIONS

**Sofia Esteban‐Serna:** Conceptualization; investigation; writing – original draft; project administration. **Sander Granneman**: Conceptualization; investigation; funding acquisition; writing – original draft; project administration; supervision. **Hugh McCaughan:** Data curation; formal analysis.

## ETHICS STATEMENT

This review does not involve human subjects, patient medical records or animals.

## Data Availability

The data that support the findings of this study are available from the corresponding author upon reasonable request.
